# Local infiltration anesthesia versus epidural analgesia for postoperative pain control in total knee arthroplasty: a systematic review and meta-analysis

**DOI:** 10.1186/s13018-018-0770-9

**Published:** 2018-05-16

**Authors:** Chen Li, Ji Qu, Su Pan, Yang Qu

**Affiliations:** Department of orthopaedics, The Second Hospital of Jilin University, Changchun, Jilin Province 130041 People’s Republic of China

**Keywords:** Local infiltration anesthesia, Total knee arthroplasty, Pain control, Meta-analysis

## Abstract

**Background:**

We performed a systematic review and meta-analysis of randomized controlled trials (RCTs) to evaluate the efficacy and safety of local infiltration anesthesia (LIA) versus epidural analgesia (EPA) for postoperative pain control in total knee arthroplasty (TKA).

**Methods:**

In December 2017, a systematic computer-based search was conducted in Pubmed, EMBASE, Web of Science, and Cochrane Database of Systematic Reviews. RCTs of patients prepared for spine surgery that compared LIA versus EPA for postoperative pain control in TKA were retrieved. The primary endpoint was the VAS score with rest or mobilization at 12, 24 and 48, and 72 h. The secondary outcomes were the range of motion, the length of stay, and the occurrence of infection and nausea. After testing for publication bias and heterogeneity between studies, data were aggregated for random-effects models when necessary.

**Results:**

Seven clinical studies with 251 patients (LIA = 124, EPA = 127) were included in the meta-analysis. There was no significant difference between LIA and EPA group in terms of the VAS score with rest at 12 and 24 h. LIA was associated with a reduction of the VAS score with rest at 48 and 72 h than EPA (*P* < 0.05). There was no significant difference between the LIA group and EPA group in terms of the VAS with mobilization at 24, 48, and 72 h (*P* > 0.05). And LIA was associated with an increase of the range of motion at 24 and 48 h (*P* < 0.05) and a reduction of the length of hospital stay (*P* < 0.05). What is more, LIA was associated with a reduction of the occurrence of the nausea.

**Conclusions:**

LIA has equivalent efficacy as EPA for pain control after TKA and shows an increase of the range of motion and a reduction of the occurrence of nausea and length of hospital stay. Due to the limited number of the included studies, more high-quality RCTs are still needed to identify the long-term effects of LIA for pain control after TKA.

**Electronic supplementary material:**

The online version of this article (10.1186/s13018-018-0770-9) contains supplementary material, which is available to authorized users.

## Background

Total knee arthroplasty (TKA) is a commonly performed procedure today. It was reported that over 700,000 joint replacements are performed in the USA each year [1]. However, postoperative analgesia remains a challenging issue. More than half of the patients undergoing TKA would experience severe pain in the early postoperative period [2–4]. Appropriate pain control is a prerequisite to promoting early mobilization and functional recovery after TKA. Several options are available for postoperative pain management following TKA, but all of them have shortcomings.

Epidural analgesia consisting of a local anesthetic agent and an opioid has been a regular regimen used for postoperative analgesia after TKA. However, some studies have indicated that the benefit of epidural analgesia must be weighed against the frequency of its adverse effects such as urinary retention, hypotension, pruritus, and motor block that delays mobilization.

In recent years, there is a growing interest in the use of local infiltration analgesia (LIA) containing various constituents as a modality of postoperative pain control. The advantage of LIA is the ability to provide pain control without interfering with lower extremity motor strength, thereby allowing early mobilization of patients. Studies have shown that LIA is consistently more effective in the treatment of postoperative pain after TKA when compared with placebo. There was still controversy about which protocol is more suitable for pain control after TKA. Therefore, we searched for relevant studies and performed a meta-analysis comparing LIA versus epidural anesthesia for reducing pain intensity in TKA patients.

## Methods

This systematic review was reported according to the Preferred Reporting Items for Systematic Reviews and Meta-Analyses (PRISMA) guidelines.

### Search strategies

The following databases were searched in November 2017 without restriction of regions or publication types: Pubmed (1950–November 2017), EMBASE (1974–November 2017), Web of Science (1950–November 2017), and Cochrane Library (November 2017 Issue 3). The Mesh terms and their combinations used in the search were as follows: “local infiltration anesthesia” OR “LIA” OR “epidural anesthesia” OR “EPA” AND “((((“Arthroplasty, Replacement, Knee”[Mesh]) OR TKR) OR TKA) OR total knee replacement)” OR total knee arthroplasty. The reference lists of related reviews and original articles were searched for any relevant studies, including randomized controlled trials (RCTs) involving adult humans. Only articles originally written in English or translated into English were considered. When multiple reports describing the same sample were published, the most recent or complete report was used. Since this is a meta-analysis, no ethics committee or institutional review board approval was necessary for the study.

### Inclusion criteria and study selection

The following are included in the study: patients: adult human subjects prepared for TKA; intervention: LIA as an intervention group; comparison: EPA as a control group; outcomes: visual analogue scale (VAS) with rest or mobilization at 12, 24, 48, and 72 h, the length of hospital stay, and the occurrence of nausea and infection; and study design: RCTs. Two independent reviewers screened the title and abstracts of the identified studies after removing the duplicates of the search results. Any disagreements about the inclusion or exclusion of a study were solved by discussion or consultation with an expert. The reliability of the study selection was determined by Cohen’s kappa test, and the acceptable threshold value was set at 0.61 [5, 6].

### Data abstraction and quality assessment

A specific extraction was conducted to collect the following data from the included trials: patients’ general characteristics, country, the sample size of the control group and intervention group, and the drug and dose of LIA and EPA. Outcomes such as VAS with rest or mobilization at 12, 24, 48, and 72 h; the length of hospital stay; and the occurrence of nausea and infection were abstracted and recorded in a sheet. Postoperative pain intensity was measured by a 100-point VAS. When the numerical rating scale (NRS) was reported, it was converted to a VAS. Additionally, a 10-point VAS was converted to a 100-point VAS [7]. Data in other forms (i.e., median, interquartile range, and mean ± 95% confidence interval (CI)) were converted to the mean ± standard deviation (SD) according to the Cochrane Handbook [8]. If the data were not reported numerically, we extracted these data using “GetData Graph Digitizer” software from the published figures. All the data were extracted by two independent reviewers, and disagreements were resolved by discussion. The methodological quality of all included trials was independently assessed by two reviewers on the basis of the Cochrane Handbook for Systematic Reviews of Interventions, version 5.1.0 (http://www.cochrane-handbook.org/).

### Outcome measures and statistical analysis

Continuous outcomes (VAS with rest or mobilization at 12, 24, 48, and 72 h and the length of hospital stay) were expressed as the weighted mean differences (WMD) and respective 95% CI. Dichotomous outcomes (the occurrence of nausea and infection) were expressed as the risk ratio (RR) with 95% CI. Statistical significance was set at *P* < 0.05 to summarize the findings across the trials. The meta-analysis was calculated by Stata software, version 12.0 (Stata Corp., College Station, TX). Statistical heterogeneity was tested using the chi-squared test and *I*^2^ statistic. When there was no statistical evidence of heterogeneity (*I*^2^ < 50%, *P* > 0.1), a fixed-effects model was adopted; otherwise, a random-effect model was chosen. Publication bias was tested using funnel plots. Publication bias was assessed by funnel plot and quantitatively assessed by Begg’s test. We considered there to be no publication bias if the funnel plot was symmetrical and the *P* value was > 0.05.

### Grade of evidence

The quality of evidence for each finding was rated based on criteria established by the GRADE (Grading of Recommendations Assessment, Development and Evaluation) group [9]. The RCTs was considered as high-quality evidence, which could be downgraded to moderate, low, or very low quality for five reasons (high risk of bias, inconsistent results, indirect evidence, imprecision, and publication bias). Any disagreement was settled by discussion among the research team.

## Results

### Search results and quality assessment

**Fig. 1:**
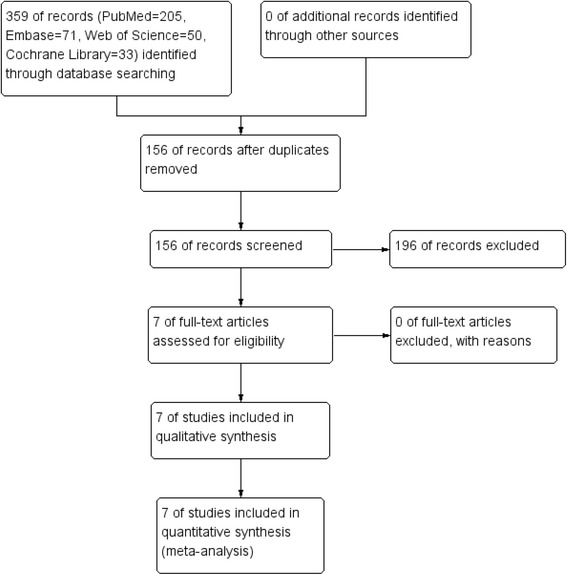
Flowchart of study search and inclusion criteria

Figure 1 details the study search and selection process. In the initial research, a total of 359 papers were identified from the electronic databases (PubMed = 205, Embase = 71, Web of Science = 50, and Cochrane Library = 33). The number of articles after duplicates had been removed by Endnote X7 software was 203. After screened the abstracts and title of these 203 studies, 196 papers were excluded because they were irrelevant or did not meet the criteria. Finally, a total of seven clinical studies involving 251 patients (LIA = 124, EPA = 127) were included in this meta-analysis [10–16]. The general characteristic of the included studies can be seen in Table 1. Included studies published from the year of 1995 to the year of 2015. And the sample of the included studies ranged from 15 to 61.

**Table 1:** The general characteristic of the included studies

Author	Country	Age (year, I/C)	Male patients (%, I/C)	LIA		EPA		Study	Follow-up
				No. of patients	Dose and methods	No. of patients	Dose and methods		
Andersen et al. 2010 [10]	Denmark	67/69	50/40	21	Combination of wound infiltration and continuous intra-articular injection postoperatively	19	Continuous epidural infusion	RCTs	30 days
Binici et al. 2014 [11]	Turkey	70.8/67.9	0/5	15	Continuous peri-articular injection postoperatively (3 ml (60 mg) lidocaine)	15	Continuous epidural infusion	RCTs	7 days
Kasture and Saraf 2015 [12]	India	67.2/67.5	12/15	40	300 ml of 0.125% bupivacaine with 5 ml ketorolac injection	35	Continuous epidural infusion (300 ml of 0.125% bupivacaine with 300 mcg fentanyl injection)	RCTs	1 month
Klasen et al. 1999 [13]	Germany	70/69	0/0	10	Single infiltration (1 mg morphine diluted in 20 ml of saline 0.9%)	10	Continuous epidural infusion (boluses of 2.5 mg of morphine)	RCTs	2 months
Spreng et al. 2010 [14]	Norway	67.2/65.8	30/41	37	Single-shot intraoperative peri-articular infiltration (ketorolac 30 mg and morphine 5 mg)	33	Continuous epidural infusion (ropivacaine 150 mg and epinephrine 0.5 mg added to isotonic saline)	RCTs	16 days
Tsukada et al. 2014 [15]	Japan	NS	NS	50	Single-shot intraoperative peri-articular infiltration	61	Continuous epidural infusion	RCTs	NS
Tsukada et al. 2015 [16]	Japan	NS	NS	37	Single-shot intraoperative peri-articular infiltration	33	Continuous epidural infusion	RCTs	6 months

**Fig. 2:**
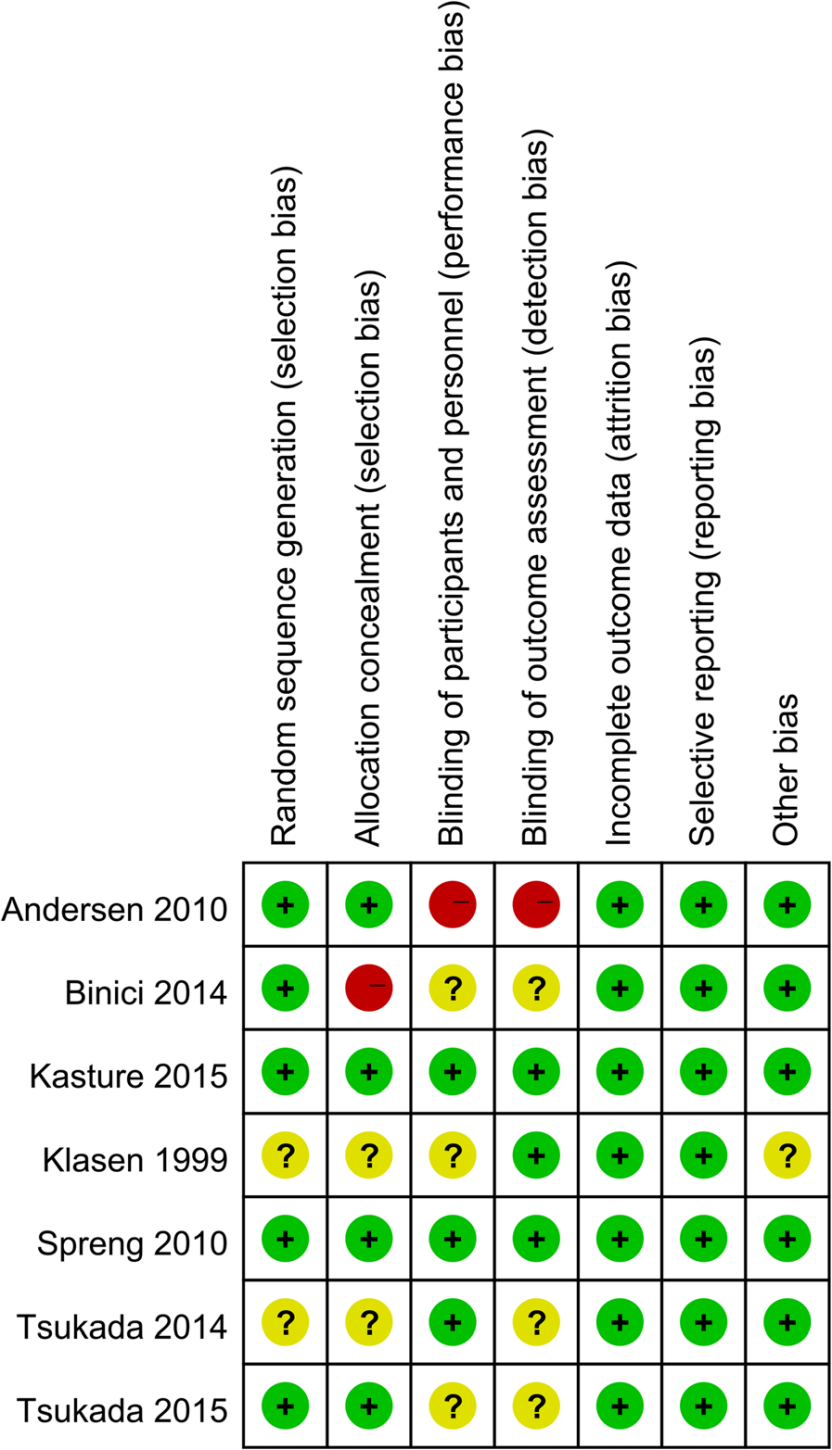
Risk of bias summary of included randomized controlled trials. +, no bias; −, bias; ?, bias unknown

**Fig. 3:**
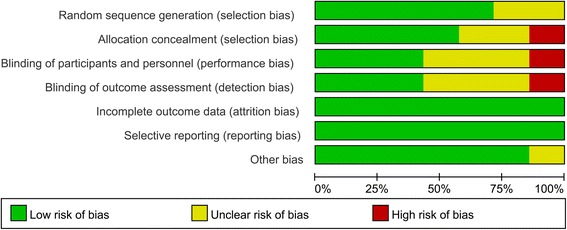
The risk of bias graph

The risk of bias summary and risk of bias graph can be seen in Figs. 2 and 3. The random sequence generation was low risk of bias in five studies, and two studies were unclear risk of bias. The allocation concealment were low risk of bias in four studies, high risk of bias in one study, and unclear risk of bias in the rest of the studies. The blinding of participants and outcomes assessment were unclear risk of bias in four studies and the selection reporting were low risk of bias in all of the included studies.

### VAS with rest at 12, 24, 48, and 72 h

**Fig. 4:**
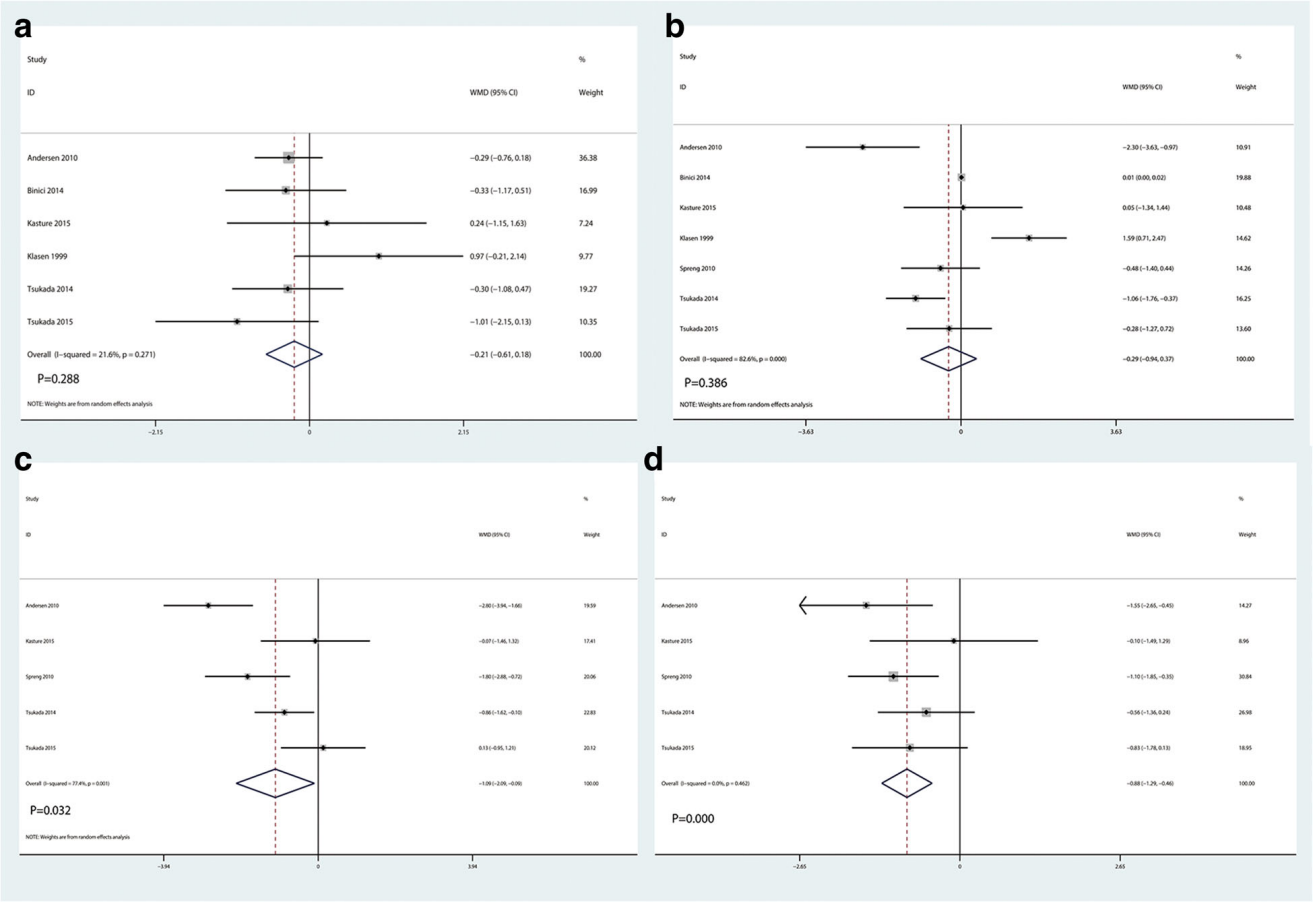
Forest plots of the included studies comparing the VAS with rest at 12 h (**a**), 24 h (**b**), 48 h (**c**), and 72 h (**d**)

Postoperative VAS scores with rest at 12 h were reported in six studies, and the pooled results indicated that there was no significant difference between the LIA and EPA group in terms of the VAS score with rest at 12 h (WMD = − 0.21, 95% CI − 0.61, 0.18, *P* = 0.288, Fig. 4a). There were a total of seven clinical studies performing the results of VAS score with rest at 24 h. Results indicated that there was no significant difference between the two groups as regard to the VAS score with rest at 24 h (WMD = − 0.29, 95% CI − 0.94, 0.37, *P* = 0.386, Fig. 4b). VAS score with rest at 48 h were available in five studies, and pooled results indicated that LIA was associated with a reduction of the VAS score with rest at 48 h than EPA (WMD = − 1.09, 95% CI − 2.09, − 0.09, *P* = 0.032, Fig. 4c). VAS score with rest at 72 h were available in five studies, and pooled results indicated that LIA was associated with a reduction of the VAS score with rest at 72 h than EPA (WMD = − 0.88, 95% CI − 1.29, − 0.46, *P* = 0.000, Fig. 4d).

### VAS with mobilization at 24, 48, and 72 h

**Fig. 5:**
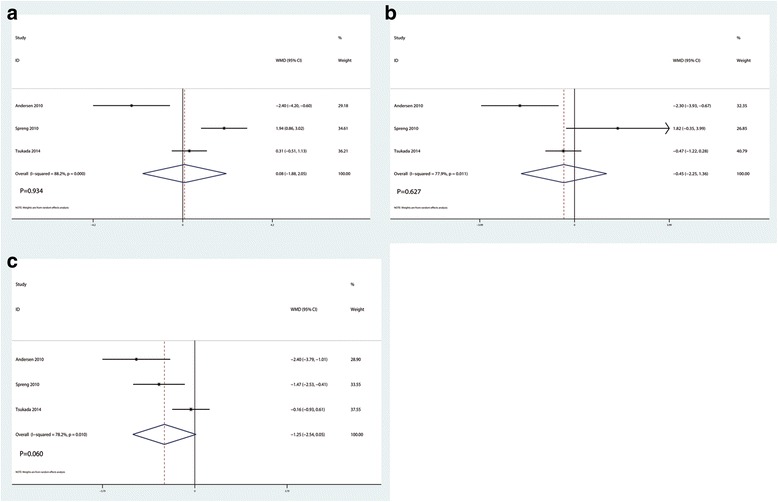
Forest plots of the included studies comparing the VAS with mobilization at 24 h (**a**), 48 h (**b**), and 72 h (**c**)

There were no significant differences between the LIA group versus EPA group in VAS score with mobilization at 24 h (WMD = 0.08, 95% CI − 1.88, 2.05, *P* = 0.934, Fig. 5a), 48 h (WMD = − 0.45, 95% CI − 2.25, 1.36, *P* = 0.627, Fig. 5b) and 72 h (WMD = − 1.25, 95% CI − 2.54, 0.05, *P* = 0.060, Fig. 5c).

### Range of motion at 24, 48, and 72 h

**Fig. 6:**
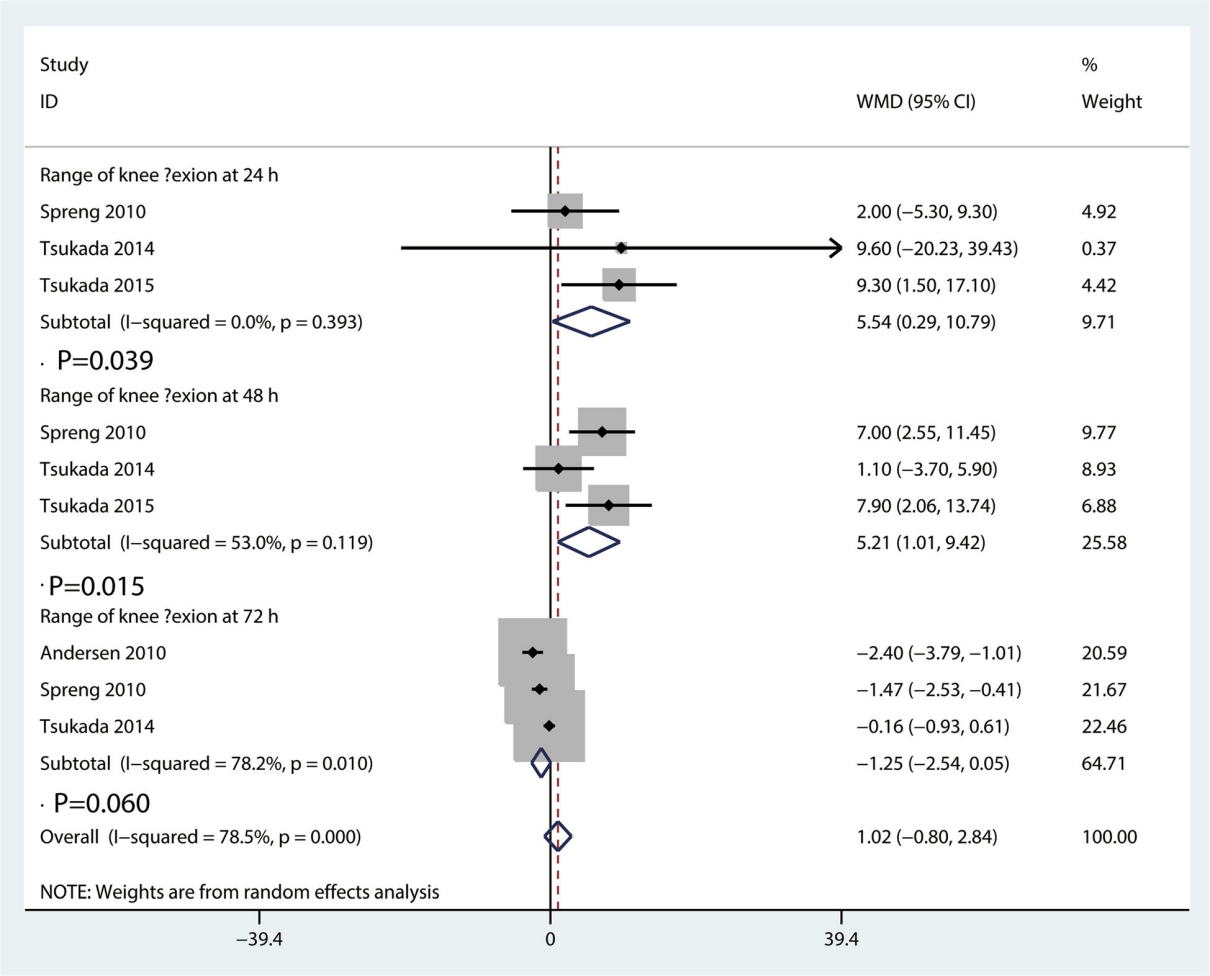
Forest plots of the included studies comparing the range of motion at 24, 48, and 72 h

Meta-analysis results indicated that LIA was associated with an increase of the range of motion than EPA at 24 h (WMD = 5.44, 95% CI 0.29, 10.79, *P* = 0.039, Fig. 6) and 48 h (WMD = 5.21, 95% CI 1.01, 9.42, *P* = 0.015, Fig. 6). The pooled results indicated that there was no significant difference between LIA group and EPA in terms of range of motion at 72 h (WMD = − 1.25, 95% CI − 2.54, 0.05, *P* = 0.060, Fig. 6).

### Length of hospital stay

**Fig. 7:**
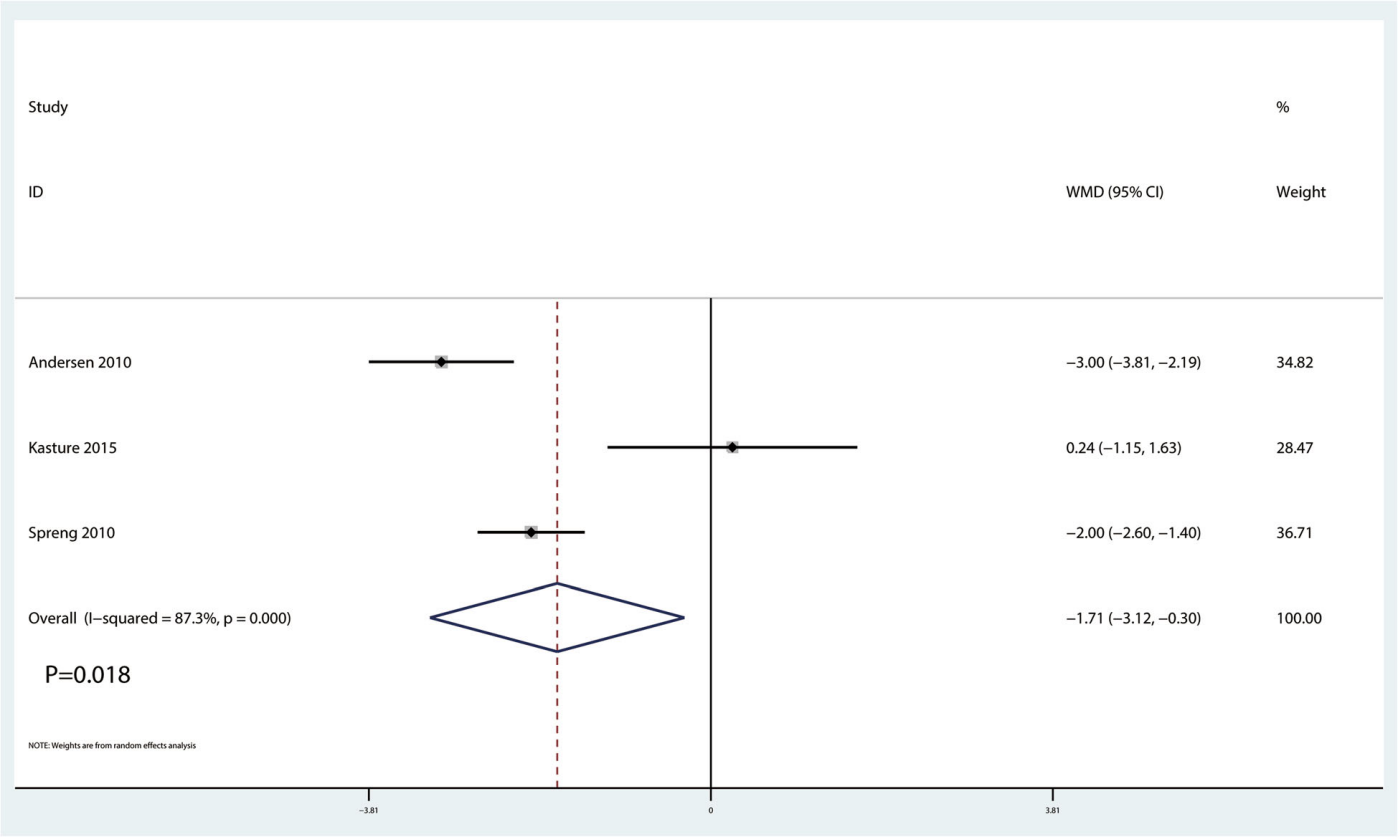
Forest plots of the included studies comparing the length of hospital stay between the two groups

Meta-analysis results indicated that LIA was associated with a reduction of the LOS than EPA (WMD = − 1.71, 95% CI − 3.12, − 0.30, *P* = 0.018, Fig. 7).

### The occurrence of nausea and infection

**Fig. 8:**
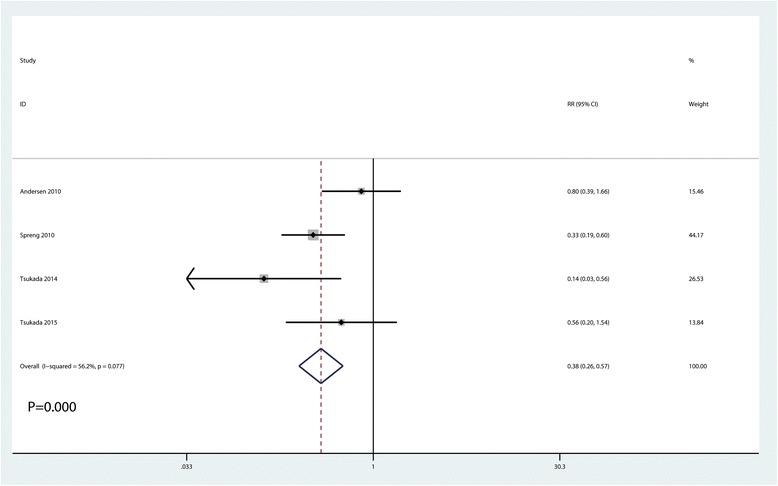
Forest plots of the included studies comparing the occurrence of nausea between the two groups

**Fig. 9:**
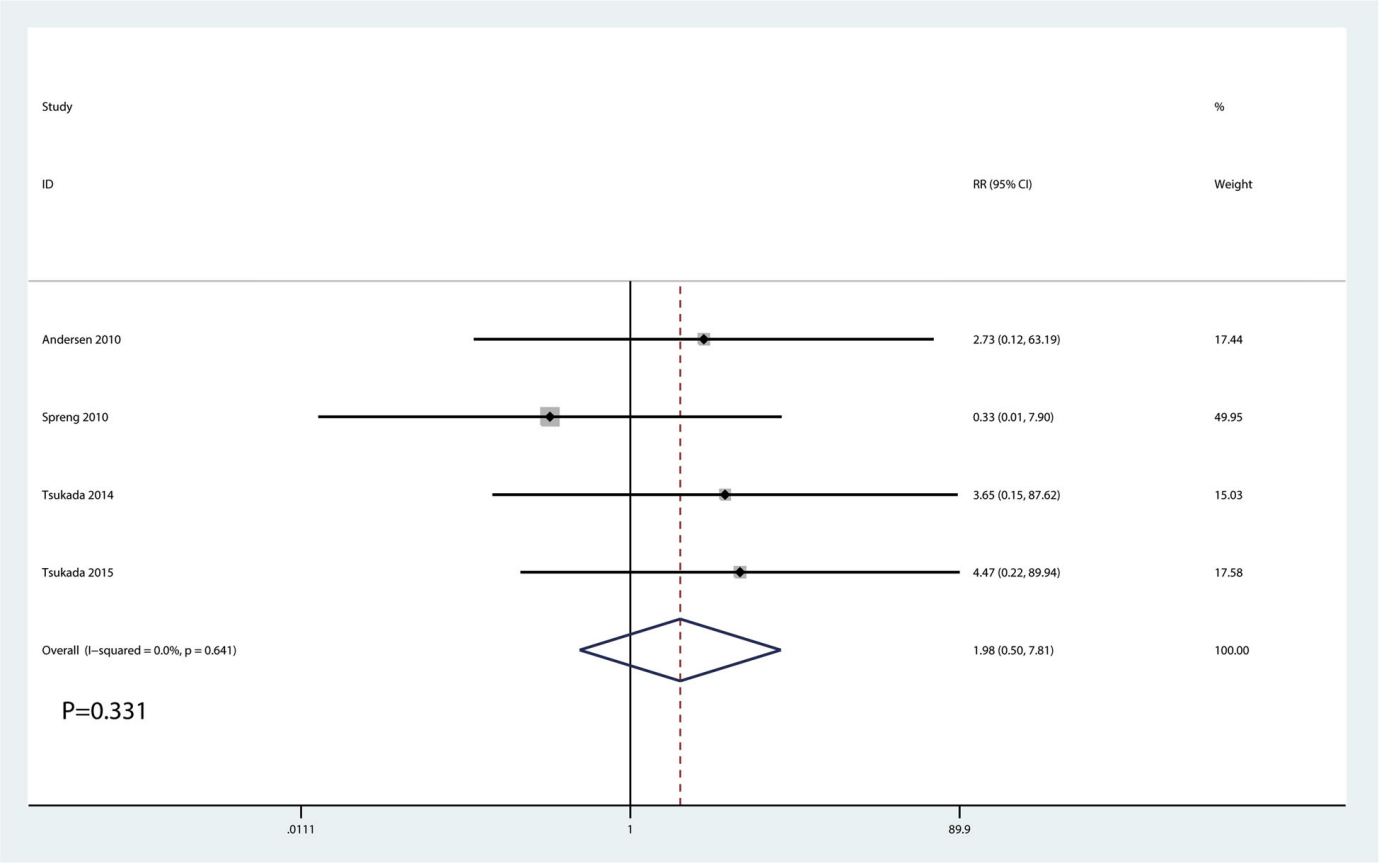
Forest plots of the included studies comparing the occurrence of infection between the two groups

Meta-analysis results indicated that LIA was associated with a reduction of the occurrence of nausea than EPA (RR = 0.38, 95% CI 0.26, 0.57, *P* = 0.000, Fig. 8). There was no significant difference between the LIA group and EPA group in terms of the occurrence of infection (RR = 1.98, 95% CI 0.50, 7.81, *P* = 0.331, Fig. 9).

### Grade of evidence

Grade of evidence was low in VAS with rest or mobilization at 12, 24, 48, and 72 h. And the grade of evidence was high in the length of hospital stay and the occurrence of nausea and infection. Grade of evidence was middle in the range of motion at 24, 48, and 72 h.

### Publication bias, sensitivity analysis, and subgroup analysis

**Fig. 10:**
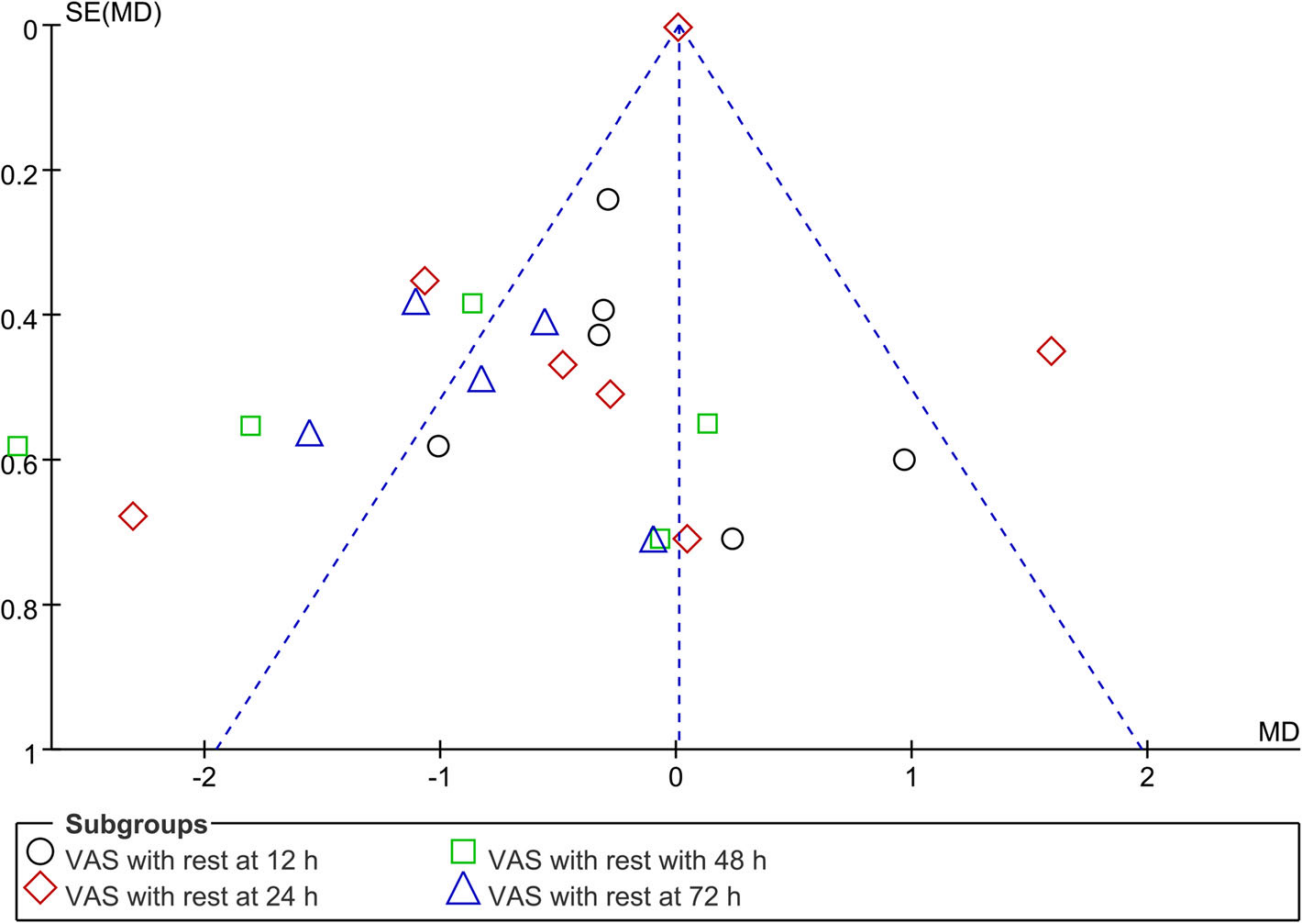
Funnel plot that comparing the VAS with rest at 12, 24, 48, and 72 h

**Fig. 11:**
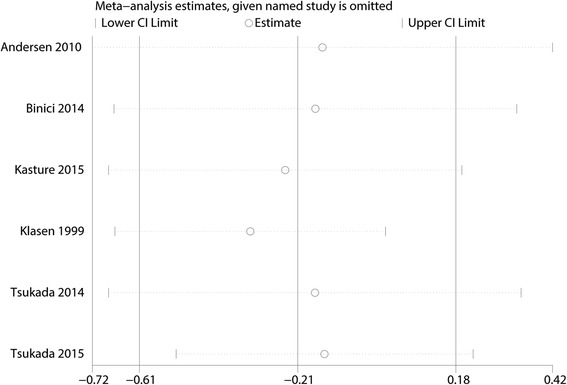
Sensitivity analysis of the VAS with rest at 12 h

Funnel plot analyses on VAS with rest at 12, 24, 48, and 72 h demonstrated symmetry, suggesting that bias was minimal (Fig. 10). Firstly, we applied leave-out method by excluding some studies to reduce between-study heterogeneity, thereby making a more robust conclusion (Fig. 11). The conclusions remained unchanged in all outcomes, suggesting the stability of our meta-analysis. We further conducted a subgroup analysis for VAS scores (single-shot LIA or continuous LIA). Subgroup result of VAS with rest at 12, 24, 48, and 72 h was shown in Additional file 1: Figure S1. Subgroup difference was found in VAS with rest at 48 h (single-shot LIA, WMD = − 2.80, 95% CI − 3.94, − 1.66, *P* = 0.000; continuous LIA, WMD = − 0.69, 95% CI − 1.51, 0.12, *P* = 0.093). VAS with mobilization at 24, 48, and 72 h was shown in Additional file 2: Figure S2. Subgroup difference was found in VAS with mobilization at 48 and 72 h.

## Discussion

### Summary of the main finding

Current meta-analysis indicated that LIA has an equivalent efficacy for pain relieving with rest of mobilization at early period and late period than EPA after TKA. LIA was associated with an increase of the range of motion than EPA at an early period after TKA. And LIA was associated with a reduction of the occurrence of nausea and the length of hospital stay than EPA. There was no significant difference between the occurrence of infection. Yan et al. [17] conducted a recent meta-analysis of RCTs showing that LIA achieves better analgesic effects comparing with EPA. However, it contained some methodological shortcomings, errors in inclusion criteria (TKA and total hip arthroplasty (THA)) and data extraction, and high heterogeneity. Considering all these issues, it is impossible to give clear advice on which method to adopt.

Pain intensity was measured as VAS score at 12, 24, 48, and 72 h after TKA. Choi et al. [18] performed a Cochrane review and revealed that EPA may be useful for postoperative pain relief at early (four to 6 h) postoperative period following TKA. These results were in accordance with our main results. Jiménez-Almonte et al. [19] using the novel statistical network meta-analysis approach and found no differences between LIA and EPA in terms of analgesia or opioid consumption 24 h after total hip arthroplasty.

With respect to the range of motion of the knee, we found that LIA was superior than EPA in the early period after TKA. These may not be an indicator of favorable pain control in the LIA group. It is more likely attributed to the absence of motor block. As a result, early functional recovery will be strengthened by functional quadriceps [20].

Current meta-analysis also compared the occurrence of nausea and infection. Epidural analgesia consisting a variety of opioids and identified has a beneficial role in reducing pain intensity after surgeries. However, the results of many studies showed a high frequency of nausea or vomiting [21]. local anesthesia was accepted as the gold standard for postoperative pain control after the report by Bromage et al. [22]. Results shown that LIA was associated with a reduction of the occurrence of nausea than EPA. More nausea has been reported to occur with morphine than with local infiltration anesthesia [23, 24]. This can be considered a disadvantage of the epidural method. In terms of wound infection, incidences were comparable between groups and kept at low level. There was no significant difference between the occurrence of infection.

### Limitations and strengths

Our meta-analysis also has several potential limitations that should be taken into account when considering the benefits. First, our analysis comprised only seven RCTs, but one of them had a modest sample size (*n* < 100). Compared to large sample size studies, small sample size studies are inclined to overestimate the intervention effect [25], which limits the power of inference. Second, although the effect size in the funnel plot was symmetrical, we could not exclude the publication bias due to the small number of the included studies. Meanwhile, the relative short follow-up duration will underestimate the complications.

## Conclusion

Although the overall quality of the evidence can be considered “middle,” we objectively assessed the benefits and risk of LIA and EPA. Based on this meta-analysis of all currently published RCTs, the findings have important implications for the medical community, namely, that LIA is an effective alternative to provide less length of hospital stay and nausea but provides comparable level of pain relief in comparison with the EPA.

## Additional files

Additional file 1: **Figure S1.** Subgroup analysis of the VAS with mobilization at 24, 48, and 72 h (single-shot local infiltration anesthesia or continuous local infiltration anesthesia, A VAS with rest at 24 h, B VAS with rest at 48 h, and C VAS with rest at 72 h). (DOCX 1384 kb)

Additional file 2: **Figure S2.** Subgroup analysis of the VAS with rest at 12, 24, 48, and 72 h (single-shot local infiltration anesthesia or continuous local infiltration anesthesia, A VAS with rest at 12 h, B VAS with rest at 24 h, C VAS with rest at 48 h, D VAS with rest at 72 h). (DOCX 2027 kb)

## Abbreviations

CI: Confidence interval
EPA: Epidural analgesia
LIA: Local infiltration anesthesia
NRS: Numerical rating scale
PRISMA: Preferred Reporting Items for Systematic Reviews and Meta-Analyses
RCTs: Randomized controlled trials
RR: Risk ratio
SD: Standard deviation
THA: Total hip arthroplasty
TKA: Total knee arthroplasty
VAS: Visual analogue scale
WMD: Weighted mean differences
