# Supplementary material for: Local infiltration anesthesia versus epidural analgesia for postoperative pain control in total knee arthroplasty: a systematic review and meta-analysis
Source: J Orthop Surg Res. 2018 May 16;13:112. doi: 10.1186/s13018-018-0770-9 (PMC5956819; doi:10.1186/s13018-018-0770-9)
Supplement: Supplementary file 2 — Figure S2. Subgroup analysis of the VAS with rest at 12, 24, 48, and 72 h (single-shot local infiltration anesthesia or continuous local infiltration anesthesia, A VAS with rest at 12 h, B VAS with rest at 24 h, C VAS with rest at 48 h, D VAS with rest at 72 h). (DOCX 2027 kb) [file 13018_2018_770_MOESM2_ESM.docx]

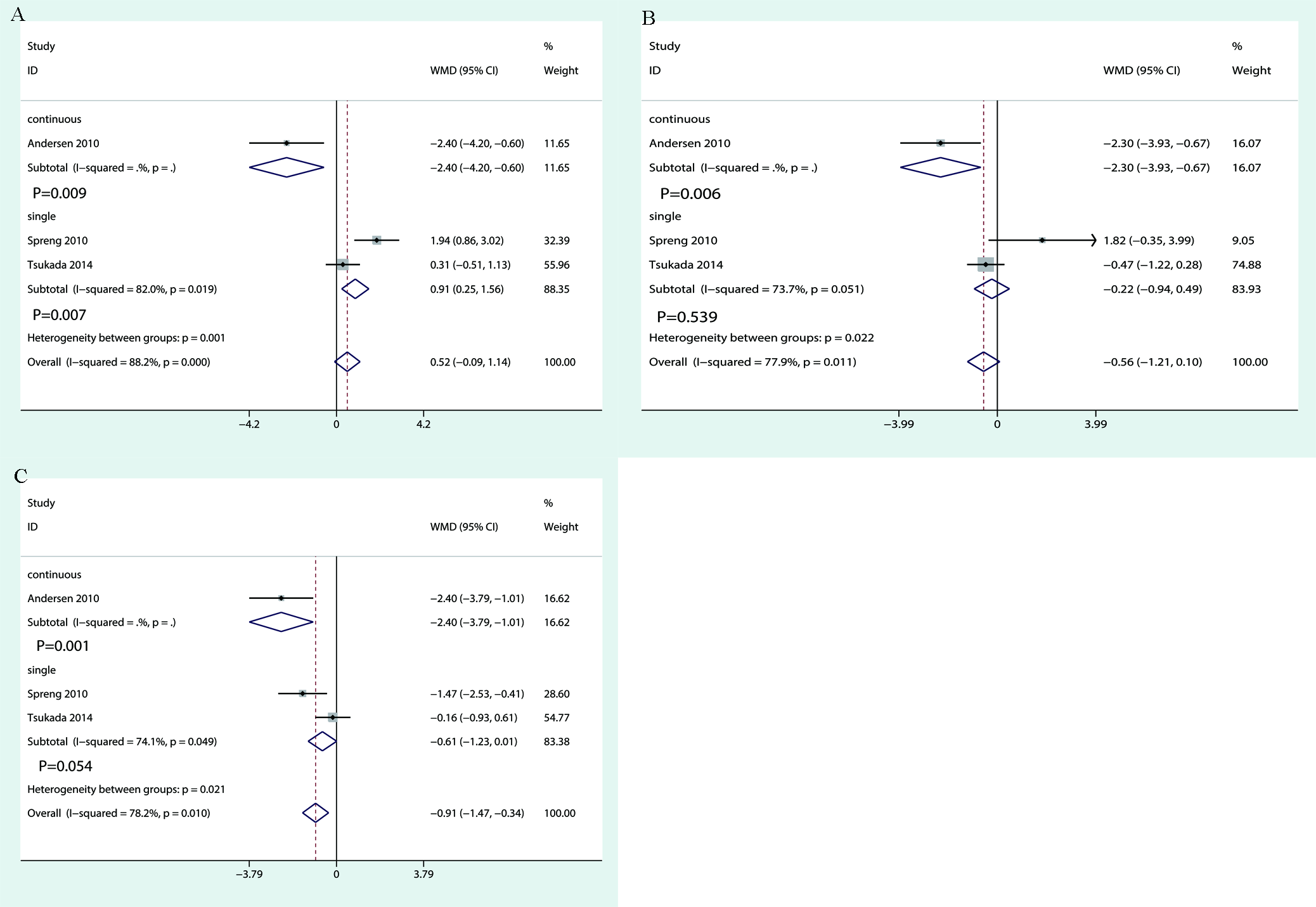


Additional file 2: Figure S2: Subgroup analysis of the VAS with rest at 12 h, 24 h, 48 h and 72 h (single shot local infiltration anesthesia or continuous local infiltration anesthesia, A, VAS with rest at 12 h, B, VAS with rest at 24 h, C, VAS with rest at 48 h, D, VAS with rest at 72 h).
